# Extended Trochanteric Osteotomy Combined with Medial Reduction Corticotomy to Correct Femoral Deformity at the Time of Revision Total Hip Arthroplasty

**DOI:** 10.2106/JBJS.OA.25.00193

**Published:** 2025-09-19

**Authors:** Diego J. Restrepo, Sergio F. Guarin Perez, Ta-Wei Tai, Matthew P. Abdel, Daniel J. Berry, Rafael J. Sierra

**Affiliations:** 1 Department of Orthopedic Surgery, Mayo Clinic, Rochester, Minnesota; 2 Department of Orthopedics, National Cheng Kung University Hospital, College of Medicine, National Cheng Kung University, Tainan, Taiwan

## Abstract

**Background:**

An extended trochanteric osteotomy (ETO) is commonly used to improve exposure and facilitate femoral component removal in revision total hip arthroplasty (THA). An additional medial corticotomy may be used in conjunction with an ETO to correct a femoral deformity, particularly varus remodeling in association with a failed femoral component. This study evaluated the outcomes of combining an ETO with a medial corticotomy in revision THA, with emphasis on implant fixation, femoral alignment, bone union, and clinical outcomes.

**Methods:**

Of the 612 ETOs performed between 2003 and 2013, 13 patients (9 men and 4 women) underwent ETO combined with a medial corticotomy to correct varus remodeling, representing 2% of all ETOs during that period. The average follow-up was 8 ± 3.5 years. The mean age at surgery was 67 ± 13.5 years. The mean body mass index was 32 ± 7.7 kg/m^2^. Radiographs were reviewed to measure preoperative and postoperative femoral deformity, time to consolidation, and femoral fixation. Clinical outcomes were evaluated using the Harris Hip Score (HHS).

**Results:**

All patients had preoperative varus femoral deformity (mean 16.3° ± 5.7°). The mean postoperative alignment was 4.3 ± 1.6° varus achieving an average correction of 12° (95% CI −15.7 to −8.3, p < 0.0001). Both the ETO and the medial corticotomy consolidated in 10 of 11 patients (91%) with available 1-year radiographs at a mean of 11 ± 7.7 months. The mean HHS improved significantly from 42 preoperatively to 82 at 5-year follow-up (p = 0.0002). Complications related to the ETO and corticotomy occurred in 4 patients (30%), including 1 intraoperative fracture, 1 postoperative greater trochanteric fracture, 1 nonunion of the medial corticotomy, and 1 postoperative wound-hematoma. All femoral components remained well fixed at final follow-up.

**Conclusion:**

The combination of ETO and medial corticotomy in revision THA effectively corrected femoral alignment in patients with a preoperative varus deformity and was associated with significant functional improvement at the final follow-up.

**Level of Evidence:**

Level IV. See Instructions for Authors for a complete description of levels of evidence.

## Introduction

Revision total hip arthroplasty (THA) in patients with femoral deformities, particularly varus remodeling, presents a surgical challenge. Proper femoral alignment is crucial for long-term success, as malalignment can lead to implant failure and poor functional outcomes. An extended trochanteric osteotomy (ETO) has been widely used to facilitate femoral component removal and improve exposure during revision THA^1-3^, especially when stems are loose and the femur has remodeled in varus alignment. In such cases, combining an ETO with a medial femoral corticotomy enables deformity correction and facilitates closure of the osteotomy around the new implant, ensuring bone-to-bone contact along longitudinal surfaces. The objective of this study was to evaluate the outcomes of combining an ETO with a medial corticotomy in revision THA, with emphasis on implant fixation, femoral alignment osteotomy union, and clinical outcomes.

## Methods

We previously reported on 612 consecutives ETOs in 596 patients between January 2003 and December 2013 at a single academic institution. All ETOs performed during this period were identified from our institution’s total joint registry that follows patients at 1, 2, and 5 years and every 5 years thereafter. Thirteen cases of an ETO combined with a medial corticotomy to correct varus femoral remodeling were identified, representing 2% of all ETOs in that decade. For these 13 patients, the revision indications included aseptic loosening in 9 patients (70%) and periprosthetic joint infection (PJI) and femoral stem fracture in 2 patients each (30%). The specific indication for adding the medial corticotomy was to correct the femoral varus deformity. Ten patients (77%) underwent revision to a modular fluted tapered stem, while 3 patients (23%) received a cylindrical fully porous-coated stem. All cases were treated with cementless revision stems.

There were 9 men and 4 women. The mean patient age was 67 ± 13.5 years. The mean body mass index (BMI) was 32 ± 7.7 kg/m^2^. The mean operative time was 225 ± 83 minutes. All patients had a minimum 2-year clinical follow-up (Table I).

**TABLE I:** Basic Characteristics of Patients Undergoing Revision Total Hip Arthroplasty with Combined Extended Trochanteric Osteotomy and Corticotomy

Demographics	Values
No. of patients	13
No. of hips	13
Age*	67 ± 13.5 (35-84)
Sex†	
Male	9 (70)
Female	4 (30)
BMI*	32 ± 7.7 (25-49)
Laterality†	
Left	7 (54)
Right	6 (46)
Operative time*	225 ± 83 (109-379)

BMI = body mass index.

* These values are given as the mean and the standard deviation, with the range in parenthesis.

† These values are given as the numbers of patients, with the percentage in parenthesis.

**Fig. 1:**
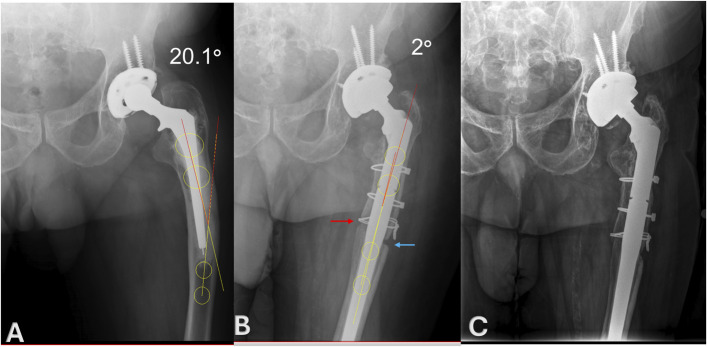
**Fig. 1-A** Preoperative anteroposterior radiographs, illustrating the anatomical axes of the proximal and distal femur. The preoperative varus angle is 20.1°. **Fig. 1-B** AP radiograph taken after revision total hip arthroplasty with combined ETO (blue arrow) and medial corticotomy (red arrow) shows a postoperative varus angle of 2°. **Fig. 1-C** AP radiographs taken 6 years postoperatively demonstrates complete healing of the medial corticotomy and maintained correction of the femoral varus deformity with stable alignment over time. No signs of implant loosening or complications are present, confirming the long-term success of the combined ETO and medial corticotomy technique. AP = anteroposterior, and ETO = extended trochanteric osteotomy.

Preoperative and immediate postoperative anteroposterior (AP) radiographs were used to measure femoral alignment. Two proximal femoral points were defined: one at the midpoint of the medial and lateral cortices at the level of the lesser trochanter and another at the midpoint of the medial and lateral cortices 1 cm distal to the lesser trochanter. A line connecting them defined the anatomical axis of the proximal femur (Fig. 1-A). Similarly, 2 distal points were identified: one at the midpoint of the cortices 1 cm distal to the femoral stem tip (or osteotomy level postoperatively) and the other 3 cm distal (Fig. 1-B). The line between them defined the distal femoral axis. The angle formed between these 2 axes represented the varus angle of the femur. Radiographic healing of the medial corticotomy was defined by bridging callus or disappearance of the osteotomy line on AP and lateral radiographs. Healing was evaluated on 1-year postoperative radiographs for 11 hips. Subsidence was assessed using immediate postoperative and 1-year AP radiographs by measuring the distance between 2 reference points: the proximal shoulder of the femoral implant and the most proximal cable. Radiographic fixation was assessed at last follow-up using standard criteria, including absence of radiolucent lines, stem migration, and evidence of bone integration.

Clinical outcomes were assessed with the Harris Hip Score (HHS)^4^ preoperatively and postoperatively at 2, 5, and 10 years. Complications were recorded from the patients’ medical records, specifically noting any nonunion of the osteotomy, wound healing complications, intraoperative or postoperative fractures, or other adverse events.

### Surgical Technique

**Fig. 2:**
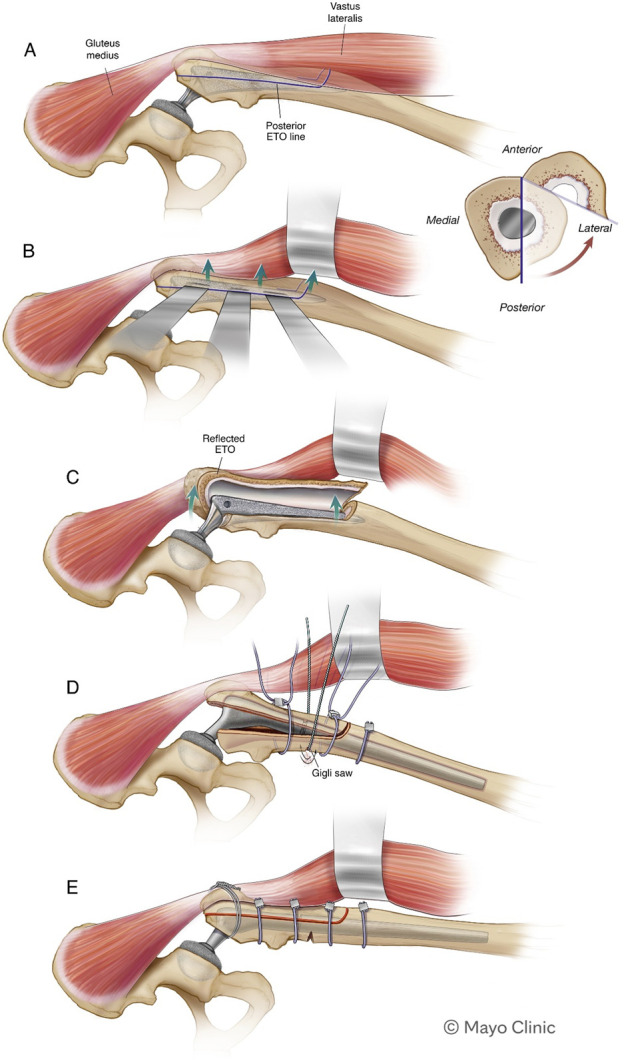
**Figs. 2-A through 2-E** Illustrations of a surgical technique combining a laterally based Paprosky ETO with a medial reduction corticotomy. ETO = extended trochanteric osteotomy.

The previous surgical incision was used in all cases. A decision to perform an ETO was made during preoperative planning; thus, in most cases, it was performed before hip dislocation and component removal. A laterally based Paprosky-style ETO technique was performed^5^. The vastus lateralis was minimally elevated off the posterior-lateral femur. An oscillating saw was used to complete the posterior longitudinal limb of the ETO, extending to 16 cm distal from the tip of the greater trochanter (Fig. 2-A). Vascularity was preserved by minimizing vastus stripping and maintaining abductor attachment to the fragment. A smaller oscillating saw was then used to perform the transverse limb of the ETO, in a beveled fashion from distal to proximal. A combination of saws and burs was used to create the distal and proximal aspects of the anterior longitudinal limb (Figs. 2-B and 2-C). A high-speed pencil-tip burr was used to complete the distal osteotomy corners in a rounded geometry. The anterior longitudinal osteotomy was completed by a controlled fracture of the remaining bone bridge, ensuring preservation of the vastus lateralis attachments and blood supply to the osteotomy fragment. The vastus lateralis and gluteus medius remained attached to the osteotomy fragment, and the lateral third of the proximal femur was reflected anteriorly. After completing the ETO, implants and cement were removed as necessary and the acetabulum was addressed as needed. A prophylactic cable was typically passed just distal to the ETO, and the femoral canal was prepared, and the final femoral stem was implanted under direct exposure of the distal femoral canal. Proximal cables were passed before hip reduction. After implantation of the final components, the hip was reduced, and the lateral ETO fragment was reduced to the distal femur and provisionally fixed with 3 cables placed strategically placed below the vastus ridge and above and below the targeted medial corticotomy site. At this point, the lateral ETO fragment would not fully oppose the medial cortex along the longitudinal osteotomy sites because of the preoperative deformity. To address this, a Gigli saw was used to osteotomize the medial cortex of the proximal femoral shaft approximately 1 to 4 cm above the distal extent of the ETO, in between the 2 distal cables (not counting a more distal prophylactic cable placed below the distal extent of the ETO (Fig. 2-D). When passing the Gigli saw, we intentionally minimized muscle stripping from the medial side of the femur. Controlled incremental tightening of the provisional cables then aided in reducing the medial bone to the lateral ETO fragment around the femoral implant (Fig. 2-E). Bone graft from femoral reaming was commonly packed along the osteotomy sites. The fascia and wound were closed in the usual manner. All patients, regardless of whether a supplementary medial corticotomy was performed, followed the same postoperative protocol: toe-touch weight-bearing for 8 weeks to protect both the ETO and femoral stem.

### Statistical Analysis

Descriptive statistics summarized cohort characteristics and outcomes. Continuous variables were reported as mean, standard deviation (SD) with range, categorical variables as frequencies, and percentages. HHS means were compared using analysis of variance, with SD and p-values provided. Continuous variables with normal distribution were compared using independent t-tests. Statistical significance was defined as p < 0.05. Analyses were performed using BlueSky software (version 10.3.4) and GraphPad Prism (version 10.0).

## Results

**Fig. 3:**
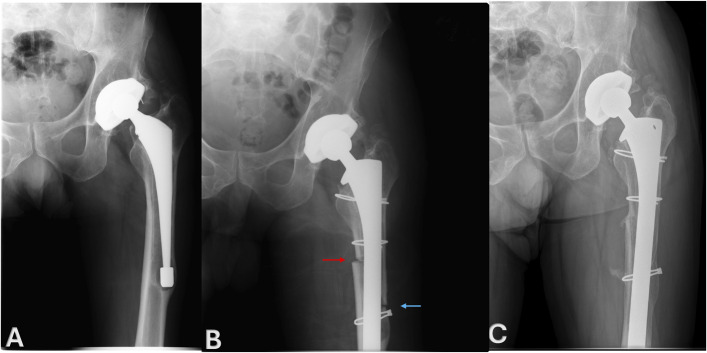
A 54-year-old man who underwent an ETO combined with a medial corticotomy for revision THA due to femoral varus deformity. **Fig. 3-A** Preoperative AP radiograph showing the varus femoral deformity. **Fig. 3-B** Immediate postoperative AP radiograph showing the revised femoral stem, the ETO (blue arrow), and the medial corticotomy (red arrow), with correction of the femoral varus deformity. **Fig. 3-C** AP radiograph 4 years postsurgery, demonstrating complete healing of both the ETO and medial corticotomy and sustained correction of the femoral varus deformity with continued alignment stability. No signs of implant loosening or complications are observed, and the femoral alignment remains well maintained, confirming the long-term success of the combined ETO and medial corticotomy procedure. AP = anteroposterior, ETO = extended trochanteric osteotomy, and THA = total hip arthroplasty.

The mean radiographic follow-up was 8 ± 3.5 years (range 2-12 years). Two patients did not have radiographic follow-up beyond 1 year. While the early postoperative images allowed for assessment of alignment, they did not show definitive signs of osteotomy healing. Although imaging was not available, both patients continued with long-term clinical follow-up and have not experienced any complications or revision at the latest follow-up. The mean preoperative femoral alignment was 16.3° ± 5.7° (range 8°-24°), and the mean postoperative alignment was 4.3° ± 1.6° (range 1.5°-7°). Thus, a mean femoral alignment correction of 12° (95% CI −15.7 to −8.3, p < 0.0001) was achieved (Fig. 3). Radiographic union of the medial corticotomy was observed in 10 of 11 patients with available 1-year radiographs (91%) at a mean of 11 ± 7.7 months. One patient experienced a nonunion of the medial corticotomy and of the distal aspect of the ETO fragment but remained asymptomatic. Five patients exhibited femoral stem subsidence between 1 and 7 mm (mean 3 ± 2.5 mm). All stems subsequently stabilized and were well fixed at the final radiographic follow-up per standard criteria.

The mean HHS increased from 42 ± 15 (range 26-48) preoperatively to 77 ± 13 (range 57-93) at 2 years postoperatively and further increased to 82 (range 65-100) at 5 years. The improvements at both 2 and 5 years were statistically significant (p = 0.0002). By 10 years, HHS data were available for 3 hips, with an average score of 55 ± 28 (range 22-75).

**Fig. 4:**
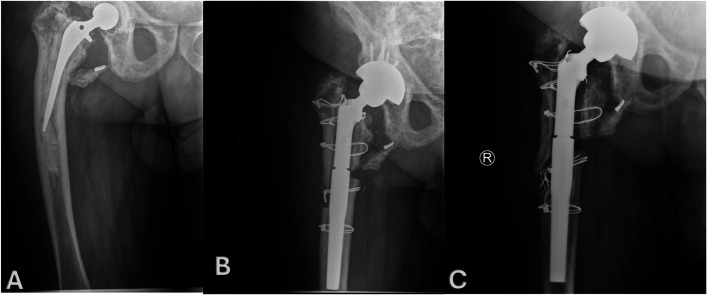
A 74-year-old man with underlying Paget disease who underwent ETO combined with medial corticotomy for revision THA due to femoral varus deformity. **Fig. 4-A** Preoperative AP radiograph demonstrating the varus femoral deformity with fracture and displaced right lesser trochanter containing a broken screw. **Fig. 4-B** One-month postoperative AP radiograph showing the ETO and medial corticotomy with correction of femoral varus deformity. Note the persistent gaps between the trochanteric fragment and the femoral shaft. **Fig. 4-C** Twelve-year postoperative AP radiograph showing the nonunion of medial corticotomy, ETO, and trochanteric fragments. AP = anteroposterior, ETO = extended trochanteric osteotomy, and THA = total hip arthroplasty.

Complications occurred in 4 patients (30.8%). As noted above, one patient with Paget disease experienced nonunion of the medial corticotomy, along with a nonunion of the ETO (Fig. 4). Another patient sustained an intraoperative fracture of the extended greater trochanteric osteotomy fragment, which occurred during femoral preparation. The medial cortex remained intact, and the fracture did not compromise implant stability. One patient developed a postoperative greater trochanteric fracture, which healed uneventfully. Another required surgical evacuation of postoperative hematoma and ETO refixation due to persistent motion.

## Discussion

Performing ETOs for revision THA in complex cases has become common. ETOs facilitate the removal of well-fixed femoral components in both septic and aseptic scenarios and provide direct access for distal cement removal and extraction of complex intracortical hardware^1,4,6-9^. There is a paucity of studies addressing the combined use of an ETO with a medial femoral corticotomy to access to the canal for implant removal while simultaneously correcting a femoral deformity during the revision procedure. To the best of our knowledge, this is the first series to comprehensively evaluate this combined technique in revision THA. We report satisfactory outcomes, demonstrating correction of the femoral varus deformity in all patients and successful osteotomy healing in 10 of 11 patients with radiographic follow-up.

Femoral deformity was corrected to a mean alignment of 4° varus, highlighting the technique’s effectiveness. A stable implant was achieved in all patients with minimal subsidence. Revising these hips without correcting the deformity might have been possible with cemented component fixation or techniques such as femoral impaction grafting; however, leaving the deformity may increase the risk of late periprosthetic fractures. The authors believe that performing the ETO allowed for implantation of an uncemented femoral component into previously uninstrumented distal bone, potentially leading to better long-term fixation. Correction of the deformity allowed for closure of the ETO after implant placement, which would not have been possible without the corrective medial cortical osteotomy.

Radiographically, the mean time to union for the medial corticotomy was 11 months, with successful union in 90% of patients with radiographic follow-up. This healing time exceeds that reported in other studies involving conventional ETO alone. Abdel et al.^1^ documented a 98% union rate at a mean of 6 months in a series of 612 ETOs. Similarly, Charity et al.^10^ reported 100% union in 18 consecutive cases at a mean of 5.8 months. Other studies have shown union rates exceeding 90%, typically achieved between 3 and 6 months in most cases^1,2,7,10-14^. The extended healing time in our series is likely due to 2 osteotomies healing simultaneously. A longer time to union should be anticipated but may be minimized by maintaining good soft-tissue attachments to the bony fragments. In this series, we deliberately made the medial corticotomy 1 to 4 cm proximal to the distal extent of the ETO, providing a “step cut” configuration which provided extrarotational stability and may have contributed to the high healing rate.

Our results demonstrate a significant and sustained improvement in HHS following surgery. The average HHS increased from 42 preoperatively to 82 at 5 years postoperatively. Similarly, multiple studies of patients undergoing an ETO during revision THA have reported significant improvements in mean HHS at the final follow-up^1,11-13,15-19^. These findings confirm that the functional benefits of the combined ETO and corticotomy procedure are not only immediate but also well maintained in the mid-term to long-term.

One patient in our series had a nonunion of the medial corticotomy and the ETO fragment. This patient had Paget disease, a well-known cause of bone fragility and abnormal bone remodeling^20,21^. In addition, 2 fractures occurred in our cohort: one intraoperative fracture of the ETO fragment and one postoperative trochanteric fracture, both of which healed without the need for further intervention. Previous studies have reported varying rates of intraoperative and postoperative femoral fractures (approximately 3% to 12%), like our findings^1,2,7,11-13,17,22^.

This study has several limitations. First, the small sample size limits the generalizability of our findings. However, these combined deformity cases are rare; even in a busy revision practice, only 13 such patients were treated over an 11-year period. In addition, the timing of osteotomy union may not have been precisely determined in some cases due to missing interval radiographs, which could have led to an overestimation of the true healing time. Nevertheless, the consistency of the observed outcomes—including deformity correction, implant fixation, and functional improvement—supports the robustness of our conclusions despite these limitations.

Our results demonstrate that an ETO used for component removal, combined with an additional medial corticotomy to correct a femoral varus deformity, reliably led to deformity correction and was associated with a high rate of femoral implant fixation, ETO healing, and corticotomy healing. This combined technique also achieved excellent clinical and functional outcomes with an acceptable complication rate.
